# Spike-HAR++: an energy-efficient and lightweight parallel spiking transformer for event-based human action recognition

**DOI:** 10.3389/fncom.2024.1508297

**Published:** 2024-11-26

**Authors:** Xinxu Lin, Mingxuan Liu, Hong Chen

**Affiliations:** ^1^School of Integrated Circuits, Tsinghua University, Beijing, China; ^2^State Key Laboratory of Integrated Chips and Systems, Frontier Institute of Chip and System, Fudan University, Shanghai, China; ^3^Greater Bay Area National Center of Technology Innovation, Research Institute of Tsinghua University in Shenzhen, Shenzhen, China; ^4^School of Biomedical Engineering, Tsinghua University, Beijing, China

**Keywords:** spiking neural network, human action recognition, transformer, attention branch, event-based vision

## Abstract

Event-based cameras are suitable for human action recognition (HAR) by providing movement perception with highly dynamic range, high temporal resolution, high power efficiency and low latency. Spike Neural Networks (SNNs) are naturally suited to deal with the asynchronous and sparse data from the event cameras due to their spike-based event-driven paradigm, with less power consumption compared to artificial neural networks. In this paper, we propose two end-to-end SNNs, namely Spike-HAR and Spike-HAR++, to introduce spiking transformer into event-based HAR. Spike-HAR includes two novel blocks: a spike attention branch, which enables model to focus on regions with high spike rates, reducing the impact of noise to improve the accuracy, and a parallel spike transformer block with simplified spiking self-attention mechanism, increasing computational efficiency. To better extract crucial information from high-level features, we modify the architecture of the spike attention branch and extend it in Spike-HAR to a higher dimension, proposing Spike-HAR++ to further enhance classification performance. Comprehensive experiments were conducted on four HAR datasets: SL-Animals-DVS, N-LSA64, DVS128 Gesture and DailyAction-DVS, to demonstrate the superior performance of our proposed model. Additionally, the proposed Spike-HAR and Spike-HAR++ require only 0.03 and 0.06 mJ, respectively, to process a sequence of event frames, with model sizes of only 0.7 and 1.8 M. This efficiency positions it as a promising new SNN baseline for the HAR community. Code is available at Spike-HAR++.

## 1 Introduction

Human action recognition (HAR) involves identifying and understanding human movements and has numerous applications in the real world (Sun et al., 2022). For instance, HAR can be employed in visual surveillance systems to detect hazardous activities and monitor human behavior, thereby ensuring safe operations (Lin et al., 2008). Additionally, HAR can facilitate sign language recognition (SLR). According to the latest data from the World Federation of the Deaf, there are 70 million deaf individuals worldwide using over 200 sign languages (Murray, 2018). However, learning sign language can be challenging and time-consuming, creating communication barriers for the deaf community (Hu L. et al., 2023). To address this issue, HAR for sign language recognition has been extensively researched. Most of the works focused on using RGB or gray-scale videos as input for HAR (Wang et al., 2017; Kındıroglu et al., 2022; Vázquez-Enríquez et al., 2021; Mercanoglu Sincan and Keles, 2022; Shen et al., 2024; Wang F. et al., 2023), due to their popularity and easy access. However, the recognition results of RGB-based HAR methods are inevitably influenced by the motion blur inherent to RGB cameras and static background noise (Wang et al., 2019; Wang Y. et al., 2022).

**Figure 1:**
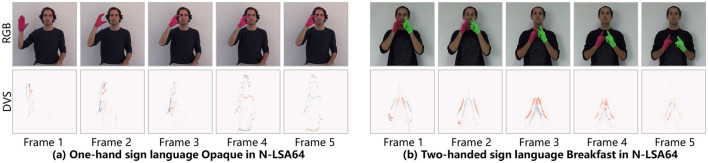
**(a)** Comparison of RGB video frames and DVS data frames for sign language Opaque (one-handed sign). **(b)** Comparison of RGB video frames and DVS data frames for sign language breakfast (two-handed sign).

As an emerging neuromorphic sensor, the event camera detects changes in brightness for each pixel independently, generating an event stream asynchronously and sparsely. The difference between RGB video frames [from LSA64 (Ronchetti et al., 2023)] and DVS event frames (from N-LSA64) is shown in Figure 1. The event camera features high temporal resolution, low latency, low power consumption, and a wide dynamic range (Su et al., 2022), which can effectively address issues related to motion blur and static background noise. That is, event cameras hold significant advantages in the field of HAR. The current state-of-the-art (SOTA) approaches for event-based HAR involve firstly designing event aggregation strategies converting the asynchronous output of the event camera into synfirst chronous visual frames, followed by processing using Artificial Neural Networks (ANNs) (Ghosh et al., 2019; Amir et al., 2017; Baldwin et al., 2022; Cannici et al., 2020; Innocenti et al., 2021; Sabater et al., 2022), which require considerable computational power, posing challenges for deployment on edge devices.

As third-generation neural networks, Spike Neural Networks (SNNs) are designed with biological plausibility, mimicking the dynamics of brain neurons to encode and transmit information in the form of spikes (Maass, 1997). Compared to ANNs, the event-driven nature of SNNs significantly reduces energy consumption when running on neuromorphic chips (Zhang et al., 2023, 2021). However, current SNN-based HAR tasks still face challenges of lack of datasets and low recognition accuracy (Shi et al., 2023).

**Figure 2:**
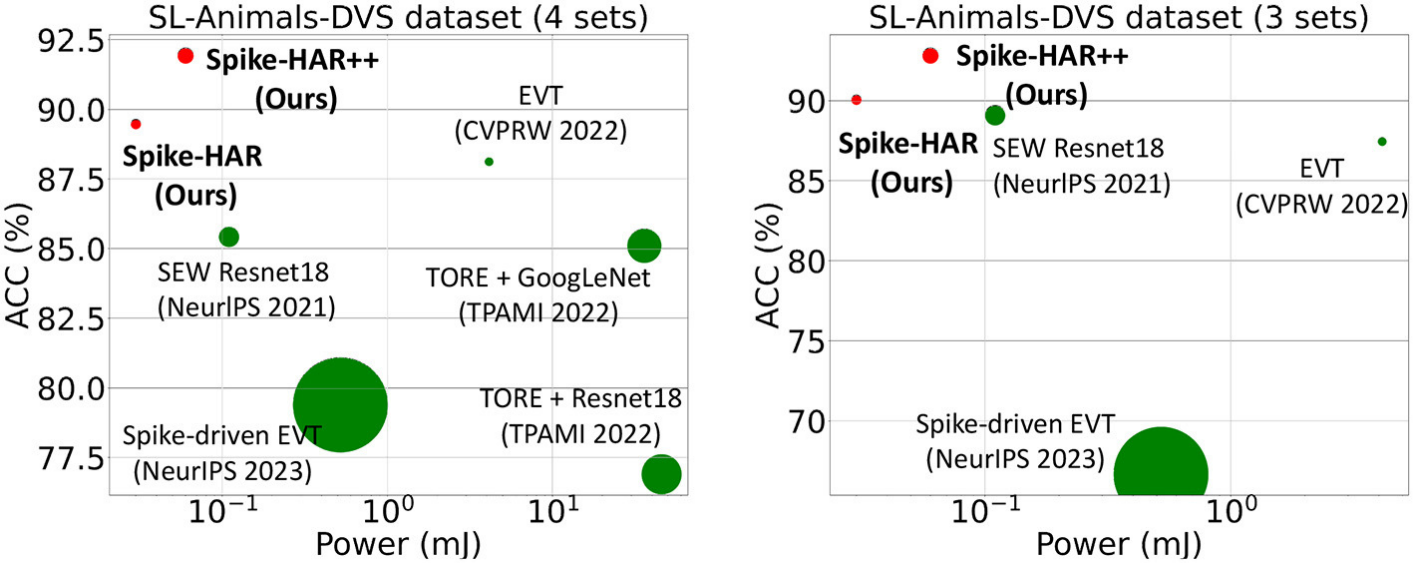
Accuracy vs. inference energy of different neural methods implemented in Intel Stratix 10 TX (Corporation, 2023) (for ANNs) or ROLLS (Qiao et al., 2015) (for SNNs). The size of the markers denotes the number of parameters.

In this paper, we propose two models, Spike-HAR and Spike-HAR++, to simultaneously reduce power consumption and enhance recognition accuracy in event-based HAR. Spike-HAR integrates a patch embedding (PE) block, parallel transformer blocks, a spike attention branch, and a classification head. To further improve performance, we modify the architecture and position of spike attention branch in Spike-HAR according to the Hu et al. (2024) and extend it to a higher dimension, proposing Spike-HAR++, which enables better extraction of crucial information from high-level features. As illustrated in Figure 2, experiments on the SL-Animals-DVS dataset (Vasudevan et al., 2022) demonstrate that both models significantly outperform other event-based HAR systems while maintaining lower levels of power consumption.

This paper is an extended version of our prior work (Lin et al., 2024) accepted by BMVC 2024. The main differences with the conference version are as follows: (1) besides the Spike-HAR based on the Parallel Spiking Transformer (referred to as Spike-SLR in the BMVC version), we newly propose Spike-HAR++, which is better suited for recognizing long-duration actions; (2) the application scope of the models are extended from sign language recognition to human action recognition, with comprehensive testing conducted on two additional datasets: DVS128 Gesture (Amir et al., 2017) and DailyAction-DVS (Liu et al., 2021), achieving SOTA performance; (3) a detailed overview about traditional ANN-based and SNN-based HAR methods, as well as the development of spiking transformers are discussed in the related work. To sum up, the main contributions of this paper are listed:

(1) We propose the Spike-HAR family, i.e., Spike-HAR and Spike-HAR++, which mainly consists of a powerful parallel spike transformer block. To the best of our knowledge, it is the first spiking transformer specifically designed for event-based HAR. To enhance the model's spatio-temporal attention to fine-grained action features while maintaining energy efficiency and a lightweight design, we employ a parallel spiking transformer. In this architecture, multi-layer perceptrons (MLPs) and simplified attention sub-modules (CB-S3A) operate in parallel to improve overall efficiency.

(2) We first introduce attention mask mechanisms into SNNs and incorporate a spike attention branch in our model to extract key regions from the input event streams. Additionally, we improve the attention operation for Spike-HAR++, utilizing high-dimensional features extracted through a patch embedding (PE) block to accommodate the recognition of long-duration actions. Experiments demonstrate that, although the parameter count and power consumption of Spike-HAR++ increase slightly, the accuracy of HAR improves significantly.

(3) Experimental results on the public datasets SL-Animals-DVS (Vasudevan et al., 2022), N-LSA64 (Ronchetti et al., 2023) [converted using the v2e (Hu et al., 2021) method], DVS128 Gesture (Amir et al., 2017), and DailyAction-DVS (Liu et al., 2021) show that the proposed Spike-HAR family effectively balances model size and recognition accuracy. Specifically, the proposed Spike-HAR and Spike-HAR++ require only 0.03 and 0.06 mJ, respectively, to process a sequence of event frames, with model size of just 0.7 and 1.8 M.

In the rest of the paper, Section 2 presents the related work on event-based HAR and spiking transformers. Section 3 begins with an overview of the overall architecture of Spike-HAR and Spike-HAR++, followed by a detailed description of each model component. Section 4 introduces four HAR benchmark datasets and evaluation metrics, along with rigorous ablation studies, visualizations, and performance evaluations of the proposed models. Finally, Section 5 concludes the paper.

## 2 Related work

### 2.1 Event-based human action recognition

Human action recognition aims to assign labels to various human behaviors and has wide applications in fields such as visual surveillance systems (Prati et al., 2019; Lin et al., 2008; Nasir et al., 2022), sign language recognition (Lin et al., 2024), autonomous navigation systems (Wang Q. et al., 2022), and video retrieval (Sahoo et al., 2020). Traditional HAR methods commonly use RGB or grayscale video as input due to their accessibility. However, HAR based on RGB modalities is not robust to illumination changes and is susceptible to motion artifacts (Sun et al., 2022). Additionally, the large data size of RGB videos results in high computational costs when modeling spatiotemporal context for HAR. To address above problem, alternative data forms for HAR have emerged, such as skeleton (Wang and Yan, 2023), depth (Sahoo et al., 2020), infrared sequences (Ding et al., 2022), point clouds (Yu et al., 2022), and event streams. This study focuses on event-based HAR, as event cameras offer high dynamic range, low latency, low power consumption, and eliminate motion blur, making them well-suited for HAR. Furthermore, the captured frames typically lack background information, which aids in action understanding.

The methods for event-based HAR can be primarily categorized into ANN-based and SNN-based (Gao et al., 2023). For ANN-based methods, representative studies mainly utilize 3D CNNs or transformers to learn features in both spatial and temporal domains, thereby aggregating information from adjacent frames. For example, Wang et al. (2024) presented a novel event stream-based action recognition model called EVMamba, which integrates a spatial plane multi-directional scanning mechanism with an innovative voxel temporal scanning mechanism to effectively extract spatio-temporal information from event streams. Acin et al. (2023) introduced VK-SITS, a new event data representation using the ResNet18 network, which outperformed other methods such as TORE (Baldwin et al., 2022) and SITS (Manderscheid et al., 2019). Additionally, Sabater et al. (2022) developed EVT, an efficient transformer model that leverages the sparsity of event data, achieving SOTA results on the SL-Animals-DVS dataset. They further improved EVT by employing a finer patch-based event data representation with richer spatio-temporal information, resulting in the introduction of the EVT+ model (Sabater et al., 2023). Gao et al. (2023) proposed the EV-ACT framework, which consists of an event voxel filtering module, a learnable multi-representation fusion module, an event-based slow-fast network, and an event-based spatio-temporal attention mechanism. This framework was tested on a new event-based HAR benchmark called THU^E − ACT^-50 and its accompanying dataset, THU^E − ACT^-50-CHL. Although ANN-based methods have achieved SOTA performance, they often involve high power consumption and a large number of model parameters due to the large data volume and significant information redundancy introduced by the temporal dimension, making them less suitable for edge applications in HAR. To address the problem, SNN-based methods have been proposed, leveraging their inherent temporal dynamics and energy efficiency. Specifically, Vasudevan et al. (2022) introduced the SL-Animals-DVS dataset and evaluated three types of SNNs, including SLAYER (Shrestha and Orchard, 2018), STBP (Wu et al., 2018), and DECOLLE (Kaiser et al., 2020), where the test accuracy for all models remained below 75%. Liu et al. (2021) were the first to apply motion information in SNNs for event-based action recognition, surpassing existing SNN methods on three datasets, including DailyAction-DVS. Although SNNs can achieve energy-efficient recognition, they often yield suboptimal results.

### 2.2 Spiking transformers

ANN-based transformers have achieved success in fields such as vision and natural language processing (NLP) (Achiam et al., 2023; Han et al., 2022). However, the exploration of self-attention (SA) mechanisms based on SNNs remains limited, primarily because the multiplication operations inherent in vanilla self-attention (VSA) mechanism (Vaswani et al., 2017) are incompatible with SNNs. Recently, research has increasingly focused on developing the spiking transformer, aiming at eliminating multiplication operations in SA to reduce computational complexity. Zhou et al. (2022) were the first to introduce spiking transformer model, termed Spikformer, which utilizes spike-based Query, Key, and Value to model sparse visual features, thereby avoiding softmax computations. Subsequently, Yao et al. (2024b) introduced the Spike-driven Transformer, which enhances the spiking self-attention (SSA) mechanism in the Spikeformer. They proposed a Spike Driven Self-Attention (SDSA) that utilizes only masking and addition to implement the SA mechanism, reducing the computational complexity from *O*(*ND*^2^) to *O*(*ND*). Wang Z. et al. (2023) introduced a novel Masked Spike Transformer (MST) framework, incorporating a Random Spike Masking (RSM) method, to further prune redundant spikes and reduce energy consumption without sacrificing performance. These exploration of spiking transformers enhance the learning capabilities of SNNs, enabling their application in various fields such as audio-visual classification, human pose tracking, and remote photoplethysmography (Guo et al., 2023; Zou et al., 2023; Liu et al., 2024). However, there is a lack of spiking transformers specifically designed for event-based HAR. We are the first to propose Spike-HAR (Lin et al., 2024), which is primarily composed of an energy-efficient parallel spiking transformer and has been tested on two DVS sign language datasets. Subsequently, SVFormer (Yu et al., 2024) was introduced as a direct training spiking transformer for efficient video action recognition, but it mainly focuses on RGB-based HAR. Wang X. et al. (2023) proposed a model called SSTFormer, which bridges SNNs and memory support transformers. However, SSTFormer is a hybrid SNN-ANN network requires both RGB frames and event streams to perform HAR. Therefore, dedicated spiking transformer models for event-based HAR still require further investigation and validation on larger-scale datasets.

## 3 Methodology

The proposed Spike-HAR and Spike-HAR++ apply the spiking transformer to HAR tasks. We utilize the SNNs algorithm provided in the SpikingJelly platform (Fang et al., 2023), employing the Leak Integrate and Fire (LIF) (Stein and Hodgkin, 1967) neural model for constructing the spiking neuron layers. LIF can be simply expressed by the following equation:


(1) $$\begin{array}{l} H\left[t\right]=V\left[t-1\right]+\frac{1}{\tau }\left(X\left[t\right]-\left(V\left[t-1\right]-V_{reset}\right)\right) \end{array}$$

(2) $$\begin{array}{l} S\left[t\right]=\Theta \left(H\left[t\right]-V_{th}\right) \end{array}$$

(3) $$\begin{array}{l} V\left[t\right]=H\left[t\right]\left(1-S\left[t\right]\right)+V_{reset}S\left[t\right] \end{array}$$

where *t* denotes the timestep, τ represents the membrane time constant, *X*[*t*] donates the synaptic input current at time step *t*, and *H*[*t*] is the neuron's membrane potential post charging and pre-spike, derived by integrating the input current. The spike occurrence at time *t*, denoted by *S*[*t*], is determined by the Heaviside step function Θ, which outputs a spike (value of 1) when *H*[*t*] surpasses the firing threshold *V*_*th*_, indicating an action potential. *V*[*t*] represents the membrane potential after spiking, which equals to *H*[*t*] if no spike occurs and otherwise reset to *V*_*reset*_.

### 3.1 Overall architecture

**Figure 3:**
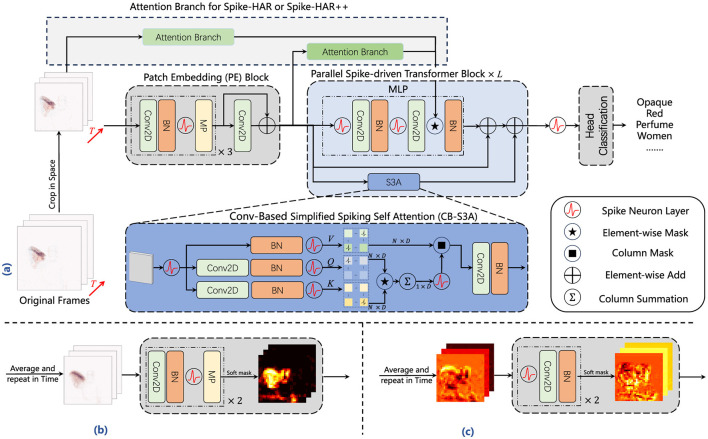
Framework of Spike-HAR and Spike-HAR++. We follow the network structure in Yao et al. (2024b). It consists of an SNN-based patch embedding (PE) block, several parallel spike-driven transformer blocks, a spike attention branch, and a SNN-based predictor head. **(a)** Architecture of Spike-HAR and Spike-HAR++. **(b)** Attention branch for Spike-HAR. **(c)** Attention branch for Spike-HAR++.

To lighten the models, we adopt less weight parameters and simpler model structures. The parameters of Spike-HAR and Spike-HAR++ is provided in Table 6, which are less than most models. In terms of model structure, Spike-HAR and Spike-HAR++ use a more simplified data preprocessing layer compared to the Spiking Transformer. And in both models we only use two MLP layers. Figure 3a illustrates the structure of Spike-HAR and Spike-HAR++, both of which consist of four main components: the patch embedding (PE) block, the parallel spike-driven transformer block, the spike attention branch, and the classification head. The PE block extracts spatio-temporal representations from the input DVS frames, while the CB-S3A module in the transformer and the spike firing rate map in the spike attention branch direct the model's focus toward key features. The final prediction head maps these features to possible sign language expressions.

Given a 2D DVS frames sequence $I_{0}\in {\mathbb{R}}^{T_{0}\times 2\times H_{0}\times W_{0}}$, where *T*_0_, 2, *H*_0_, *W*_0_ represent the time step, initial number of channels, height and weight respectively. Firstly we randomly select continuous event frames with a time step of *T*(*T* ≤ *T*_0_) and crop each event frame spatially to obtain the preprocessed frames (PR), denoted as *I*∈ℝ^*T*×2 × *H*×*W*^. The SNN-Based PE block, consisting of four 2D convolutional (Conv2D) layers, three batch normalization (BN) layers, three SNN layers and two max pooling (MP) layers, downsamples the input frames and partitioning them into spatio-temporal spike tokens $S_{PE}\in {\mathbb{R}}^{T\times D\times \frac{H}{4}\times \frac{W}{4}}$, where *D* represents the number of channels. Before entering the data into the parallel Spike-driven Transformer block, we use membrane potential residual connection to avoid network degradation, adding *S*_*PE*_ and the output *I*_*PE*_ of the initial three convolutional layers and resulting the input *S*_0_ of the same shape as *S*_*PE*_. Therefore, the SNN-based PE block can be written as follows:


(4) $$I=\text{PR}(I_{0})\;I_{0}\in {\mathbb{R}}^{T_{0}\times 2\times H_{0}\times W_{0}},I\in {\mathbb{R}}^{T\times 2\times H\times W}$$

(5) $$S_{PE}=\text{PE}(I)\;S_{PE}\in {\mathbb{R}}^{T\times D\times \frac{H}{4}\times \frac{W}{4}}$$

(6) $$S_{0}=I_{PE}+S_{PE}\;S_{0}\in {\mathbb{R}}^{T\times D\times \frac{H}{4}\times \frac{W}{4}}$$

Then, the spike sequence *S*_0_ is passed to the parallel spike-driven transformer blocks, which consists of a conv-based simplified spiking self-attention (CB-S3A) block and a MLP block. As the main component in Spike-HAR and Spike-HAR++, CB-S3A, which just performs the convolution operation in spike-form Query (*Q*) and Key (*K*), offers an efficient method to model the local-global information of frames without softmax. In addition, the spike fire map generated by the spike attention branch performs mask operation on the data produced by the second convolution in the MLP block, which makes model more focus on local features. The outputs of the MLP and the CB-S3A blocks are summed together, and the sum is then added to the input *S*_0_ again using membrane potential residual connection (RES). After *L* transformer blocks, the final output membrane potentials *S*_*L*_ is obtained. To obtain the pulse expression just consisting of 0 and 1, *S*_*L*_ then is passed to a spike neural layer (SN), resulting in *S*_*E*_. Finally, the *S*_*E*_ will be sent to a SNN-based classification head (SCH) to output the classification result *Y*. To summary, the output of CB-S3A, MLP and SCH can be written as follows:


(7) $$\begin{array}{l} S_{l}=\text{CB-S3A}(S_{l-1})+\text{MLP}(S_{l-1})+S_{l-1}\;\\ \;S_{l}\in {\mathbb{R}}^{T\times D\times \frac{H}{4}\times \frac{W}{4}},l=0...L \end{array}$$

(8) $$S_{E}=SN(S_{L})\;S_{E}\in {\mathbb{R}}^{T\times D\times \frac{H}{4}\times \frac{W}{4}}$$

(9) $$Y=SCH(S_{E})$$

### 3.2 Attention masks

DVS data can be influenced by noise from various sources, such as environmental background noise. As neural networks deepening, some noise may be amplified, causing the model to focus on irrelevant features. Inspired by Liu X. et al. (2020), we insert attention blocks into our model to minimize the negative impact of background noise while allowing the model to focus on the target area and local features. In order to take note of the difference among different body parts, attention mask is applied to assign higher weights to pixels with stronger spike signals, while it is also the bridge between attention appearance and the backbone network. The difference between Spike-HAR and Spike-HAR++ lies in their implementation of attention branch. In Spike-HAR, the attention map is generated by directly averaging at the frame level, while Spike-HAR++ performs information extraction at a high-dimensional feature scale. The implementation methods of both are described in detail below.

#### 3.2.1 Spike-HAR

As shown in Figure 3b, unlike the data processing operations performed in the PE block, we first perform a sum-average-repeat (SAR) operation on the data in the attention appearance. Specifically, we sum the event frames in the time dimension to combine multiple frames *I*∈ℝ^*T*×2 × *H*×*W*^ into a single frame $I_{SIN}\in {\mathbb{R}}^{1\times 2\times H\times W}$. Then, we divide the frame data by time step to obtain the average frame $I_{AVG}\in {\mathbb{R}}^{1\times 2\times Ĥ\times Ŵ}$ and replicate the *I*_*AVG*_ in the time dimension for *T* times as the input to the spike attention branch. The data $I_{E}\in {\mathbb{R}}^{T\times 2\times Ĥ\times Ŵ}$ undergoes two rounds of convolution and downsampling, followed by another SAR operation to obtain a spike fire rate map, which is then masked with the data in the MLP to facilitate communication between the branch and the backbone network as shown in Figure 3a.

#### 3.2.2 Spike-HAR++

Directly summing event frames along the temporal dimension can efficiently aggregate critical spatial information at a low cost. However, it may fail during significantly prolonged actions. To address this issue, we utilize the spatio-temporal spike tokens $S_{PE}\in {\mathbb{R}}^{T\times D\times \frac{H}{4}\times \frac{W}{4}}$ extracted from the PE block and perform a SAR operation along the temporal dimension. These tokens are subsequently fed into a new spike attention branch, where they undergo two LIF-Conv-BN operations (shown in Figure 3c), followed by averaging along the temporal dimension to produce the attention mask. By leveraging the key features extracted by the PE block, the generated multi-channel mask is more representative. Experiments (Section 4) demonstrate that, although this adjustment increases power consumption by 0.03 mJ and model complexity as the convolution block must handle a larger number of feature channels, it significantly enhances HAR accuracy across various datasets.

### 3.3 Parallel spike-driven transformer

In the previous spiking Transformer architecture (Zhou et al., 2022; Yao et al., 2024b,a), the output *U*_*out*_ of the backbone network is transformed from the input *U*_*in*_ consisting of *N* tokens with dimension *D* using two consecutive sub-blocks (one SA and one MLP) with residual connections:


(10) $$U_{\text{out}}=\alpha_{\text{FF}}\hat{U}+\beta_{\text{FF}}\text{MLP}(SN(\hat{U}))$$

(11) $$\hat{U}=\alpha_{\text{SA}}U_{\text{in}}+\beta_{\text{SA}}\text{SA}(SN(U_{\text{in}}))$$

where scalar gain weights α_FF_, β_FF_, α_SA_, β_SA_ fixed to 1 by default. In our work, to simplify the transformer block, we remove the residual connections in the MLP sub-blocks, obtaining the following output:


(12) $$\begin{array}{l} S_{\text{out}}=\alpha_{comb}S_{\text{in}}+\beta_{\text{FF}}\text{MLP}\left(\mathrm{SN}\left(S_{\text{in}}\right)\right)+\beta_{\text{SA}}\text{SA}\left(\mathrm{SN}\left(S_{\text{in}}\right)\right) \end{array}$$

with skip gain α_*comb*_ = 1, and residual gains β_FF_ = β_SA_ = 1 as default. In the submodule CB-S3A, we first input the spike signals *S*_0_ into the spike neuron layer to obtain *S*′. Then, we use 2D convolution operations to extract spatial information separately, resulting in *Q* and *K*. The acquisition of *V* does not involve convolution operations. After that, we use the spike neuron layer again to transform *Q*, *K*, and *V* into spike tensors *Q*_*S*_, *K*_*S*_, and *V*_*S*_. And the subsequent masking calculation can be represented as follows:


(13) $$\begin{array}{l} MASK\left(Q_{S},K_{S},V_{S}\right)=g\left(Q_{S},K_{S}\right)⊗V_{S}\\ \;=SN\left(SUM_{C}\left(Q_{S}⊗K_{S}\right)\right)⊗V_{S} \end{array}$$

where ⊗ denotes the Hadamard product, *g*(·) is used to compute the attention map, and *SUM*_*C*_ is used to calculate the sum of each column. The outputs of *g*(·) and *SUM*_*C*_ are row vectors of dimension *D*. Additionally, the Hadamard product between pulse tensors is equivalent to mask computation.

## 4 Experimental evaluation

### 4.1 Dataset

We evaluate our models on three public datasets, all generated by recording actions in real scenes. SL-Animals-DVS (Vasudevan et al., 2022) and DVS128 Gesture (Amir et al., 2017) were captured by a 128 × 128 pixel DVS128 camera, while DailyAction-DVS (Liu et al., 2021) was captured by a DAVIS346 camera with a spatial resolution of 346 × 260. Furthermore, we also tested our models using the N-LSA64 dataset which is transformed from LSA64 (Ronchetti et al., 2023) dataset using v2e (Hu et al., 2021) method.

#### 4.1.1 SL-Animals-DVS

In the SL-Animals-DVS dataset 59 individuals were recorded separately, and each individual performed 19 signs in sequence. Due to the fact that the recording is conducted in 4 sessions at different locations under different lighting conditions, it can be further divided into SL-Animals-DVS-4sets, which includes four shooting environments, and SL-Animals-DVS-3sets, which includes three shooting environments.

#### 4.1.2 DVS128 gesture

The DVS128 Gesture dataset comprises 1,342 recordings of 29 subjects performing 11 different actions (including one rejected class with random gestures) under three different lighting conditions.

#### 4.1.3 DailyAction-DVS

The DailyAction-DVS dataset comprises 1,440 recordings of 15 subjects acting 12 different actions, including *bend*, *climb*, *falldown*, *getup*, *jump*, *liedown*, *carrybox*, *run*, *sitdown*, *standup*, *walk* and *pickup*.The actions were captured under two lighting conditions including *naturallight* and *LEDlight*.

#### 4.1.4 N-LSA64

The N-LSA64 contains 3,200 DVS videos in which 10 non-expert subjects performed five repetitions of 64 different types of sign language. The symbols were selected from the most commonly used symbols in the LSA lexicon, including verbs and nouns. Depending on the number of hands performing the sign language, we further divide the data into N-LSA64-Right, which includes only right-hand movements, and N-LSA64-Both, which includes movements involving both hands.

We utilize a frame-based representation to preprocess an event stream (Fang et al., 2021b; Yao et al., 2021), transforming it into a sequence of event frames. Suppose the interval between two frames (i.e., temporal resolution) is *dt* and there are *T* frames (i.e., timesteps), the total length of the input event stream is *t*_*total*_ = *dt*×*T* milliseconds. After processing these frames with the proposed model, we can obtain a prediction.

### 4.2 Implementation details

We set the number of parallel spike-driven transformer block *L*= 2 in Spike-HAR and Spike-HAR++. In the DVS128 Gesture datasets, the sample length, time step, and learning rate is set as 6,000 ms, 20 and 1 × *e*^−3^ respectively. In the SL-Animals-DVS and N-LSA64 datasets, the sample length, time step, and learning rate is set as 500 ms, 10 and 1 × *e*^−4^ respectively. In the DailyAction-DVS dataset, the sample length, time step, and learning rate is set as 1,200 ms, 10 and 1 × *e*^−3^ respectively. For the training and evaluation of frame-based methods, if the number of frames contained in each event frames is larger than the timesteps *T*, we linearly sample *T* of them. Otherwise, we pad them to the length of *T* with the zero-padding operation. Spike-HAR and Spike-HAR++ are optimized with AdamW (Loshchilov and Hutter, 2017) optimizer, in a single NVIDIA GeForce RTX 3090. We set the batch size to 32 and trained for 240 epochs using the one cycle learning rate policy (Smith and Topin, 2018). As for the data augmentation, we use spatial and temporal random crop and repeat each sample within the training batch twice with different augmentations. In addition, for the N-LSA64 dataset, we divided the data into training, validation, and test sets in the ratio of 6:2:2, and evaluated the classification accuracy on the test set.

### 4.3 Comparison to the state-of-the-art models

We compare the proposed Spike-HAR and Spike-HAR++ with several relevant action recognition methods, including SNN and ANN. And the results on four datasets are shown in Tables 1–4, respectively. We can find that our proposed models outperform existing action recognition methods, indicating that our proposed models have a stronger ability to extract action information from event data. Specifically, on the SL-Animals-DVS dataset, we compare our models with existing ANN models, SNN models and a hybrid neural network that includes both ANN and SNN components. Additionally, we replace the backbone network in EVT (Sabater et al., 2022) with the Spike-Driven Transformer block (Yao et al., 2024b) to obtain Spike-Evt and conduct model training for comparative analysis. Experimental results on SL-Animals-DVS are given in Table 1, from which we can see that the accuracy of Spike-HAR++ is 3.81 and 5.37% higher than that of EVT (Sabater et al., 2022) on the dataset SL-Animals-DVS-4sets and SL-Animals-DVS-3sets, respectively. And compared to the SNN method EventRPG + SEW Resnet18 (Sun et al., 2024), the Spike-HAR++ improves the accuracy by 0.35% on the dataset SL-Animals-DVS-4sets. On the SL-Animals-DVS-3sets, the EventRPG+SEW Resnet18 achieves a higher classification accuracy of 93.30% by using complex data augmentation strategies. In contrast, Spike-HAR++ reaches a similar accuracy of 92.82% with simple data augmentation (Section 4.2) and a more lightweight backbone (Section 4.5, Spike-HAR++ vs. SEW ResNet18). On the N-LSA64-Both and N-LSA64-Right datasets, for comparison with existing methods, we adopt the same sampling and training strategies to train the SOTA ANN model EVT (Sabater et al., 2022), the baseline SNN model STBP (Wu et al., 2018), and Spike-EVT, which is constructed by replacing the EVT backbone with a spiking transformer (Yao et al., 2024b). The test results, presented in Table 2, demonstrate that Spike-HAR++ increases accuracy by 1.72% compared to EVT on the N-LSA64-Both dataset and by 5.71% compared to the Spike-driven EVT on the N-LSA64-Right dataset. Furthermore, compared to the other models, Spike-HAR and Spike-HAR++ utilize the shortest sample length of 500 ms. For the DVS128 Gesture, as can be seen in Table 3, Spike-HAR and Spike-HAR++ get the classification accuracy of 98.26 and 97.92%, respectively, outperforming other ANN and SNN methods. Finally, as shown in Table 4, we compared our results on DailyAction-DVS with state-of-the-art SNN models. Spike-HAR++ achieved the best classification performance, reaching 98.47%, using the sample length of just 1,200 ms.

**Table 1:** Classification accuracy in the SL-Animals-DVS dataset.

**Model**	**Method**	**Time step**	**Sample length**	**SL-Animals-DVS**	
				**4 sets**	**3 sets**
TORE + GoogLeNet (Baldwin et al., 2022)	ANN	\\	\	0.8510	\
TORE + ResNet18 (Baldwin et al., 2022)	ANN	\	\	0.7690	\
VoxelGrid + ResNet18 (Zhu et al., 2019)	ANN	\	\	0.8902	\
SITS + ResNet18 (Manderscheid et al., 2019)	ANN	\	\	0.7847	\
VK-SITS + ResNet18 (Acin et al., 2023)	ANN	\	\	0.7926	\
EVT (Sabater et al., 2022)	ANN	\	504 ms	0.8812	0.8745
SCTFA + 7-Layer Spiking CNN (Cai et al., 2024)	Hybrid	\	\	0.9004	\
SLAYER (Shrestha and Orchard, 2018)	SNN	300	1,500 ms	0.5430	0.6141
STBP (Wu et al., 2018)	SNN	50	1,500 ms	0.6497	0.7147
DECOLLE (Kaiser et al., 2020)	SNN	500	500 ms	0.6219	0.6219
SEW Resnet18 (Fang et al., 2021a)	SNN	16	\	0.8542	0.8909
EventDrop + SEW ResNet18 (Gu et al., 2021)	SNN	\	\	0.8633	0.8899
NDA + SEW ResNet18 (Li et al., 2022)	SNN	\	\	0.8777	0.8955
EventRPG + SEW ResNet18 (Sun et al., 2024)	SNN	\	\	**0.9159**	0.9330
Spike-Driven EVT (Yao et al., 2024b)	SNN	11	504 ms	0.7939	0.6667
**Spike-HAR (Ours)**	SNN	10	500 ms	0.8947	0.9006
**Spike-HAR++ (Ours)**	SNN	10	500 ms	0.9193	**0.9282**

Red and bold indicate the best and second best performance.

**Table 2:** Classification accuracy in the N-LSA64 dataset.

**Model**	**Method**	**Time step**	**Sample length**	**N-LSA64**	
				**Both**	**Right**
EVT (Sabater et al., 2022)	ANN	\	504 ms	0.8406	0.8214
STBP (Wu et al., 2018)	SNN	50	1,500 ms	0.5969	0.5786
Spike-driven EVT (Yao et al., 2024b)	SNN	11	504 ms	0.7266	0.8262
**Spike-HAR (Ours)**	SNN	10	500 ms	**0.8469**	**0.8690**
**Spike-HAR++ (Ours)**	SNN	10	500 ms	0.8578	0.8833

Red and bold indicate the best and second best performance.

**Table 3:** Classification accuracy in the DVS128 Gesture dataset.

**Model**	**Method**	**Time step**	**Sample length**	**DVS128 Gesture**
12 layers CNN (Amir et al., 2017)	ANN	120	120 ms	0.9260
Identify + Resnet34 (He et al., 2016)	ANN	\	\	0.9549
NDA + Resnet34 (Li et al., 2022)	ANN	\	\	0.9722
EventMix + Resnet34 (Shen et al., 2023)	ANN	\	\	0.9180
ShapeAug + Resnet34 (Bendig et al., 2024)	ANN	\	\	0.9170
EventDrop + Resnet34 (Gu et al., 2021)	ANN	\	\	0.9618
PLIF-SNN (Fang et al., 2021b)	SNN	20	6,000 ms	0.9760
Res-SNN-18 (Yao et al., 2021)	SNN	16	6,000 ms	0.9790
ASA-SNN (Yao et al., 2023)	SNN	20	6,000 ms	0.9770
Identify + SEW Resnet18 (Fang et al., 2021a)	SNN	\	\	0.9433
Eventmix + SEW Resnet18 (Shen et al., 2023)	SNN	\	\	0.9675
EventRPG + SEW Resnet18 (Sun et al., 2024)	SNN	\	\	0.9653
Identify + CSNN (Xu et al., 2018)	SNN	\	\	0.9375
NDA + CSNN (Li et al., 2022)	SNN	\	\	0.9583
EventAugmentation + CSNN (Gu et al., 2024)	SNN	\	\	0.9625
EventDrop + CSNN (Gu et al., 2021)	SNN	\	\	0.9444
**Spike-HAR (Ours)**	SNN	20	6,000 ms	0.9826
**Spike-HAR++ (Ours)**	SNN	20	6,000 ms	**0.9792**

Red and bold indicate the best and second best performance.

**Table 4:** Classification accuracy in the DailyAction-DVS dataset.

**Model**	**Method**	**Time step**	**Sample length**	**DailyAction-DVS**
Gabor-Tempotron SNN (Xiao et al., 2019)	SNN	\	\	0.6830
HMAX-SNN (Liu Q. et al., 2020)	SNN	\	\	0.7690
Motion-SNN (Liu et al., 2021)	SNN	\	\	0.9030
PLIF-SNN (Fang et al., 2021b)	SNN	36	4,320 ms	0.9250
ASA-SNN (Yao et al., 2023)	SNN	36	4,320 ms	0.9460
EHTI & MDTS-Tempotron SNN (Ding et al., 2024)	SNN	\	\	0.9608
**Spike-HAR (Ours)**	SNN	10	1,200 ms	**0.9826**
**Spike-HAR++ (Ours)**	SNN	10	1,200 ms	0.9847

Red and bold indicate the best and second best performance.

### 4.4 Ablation study

**Figure 4:**
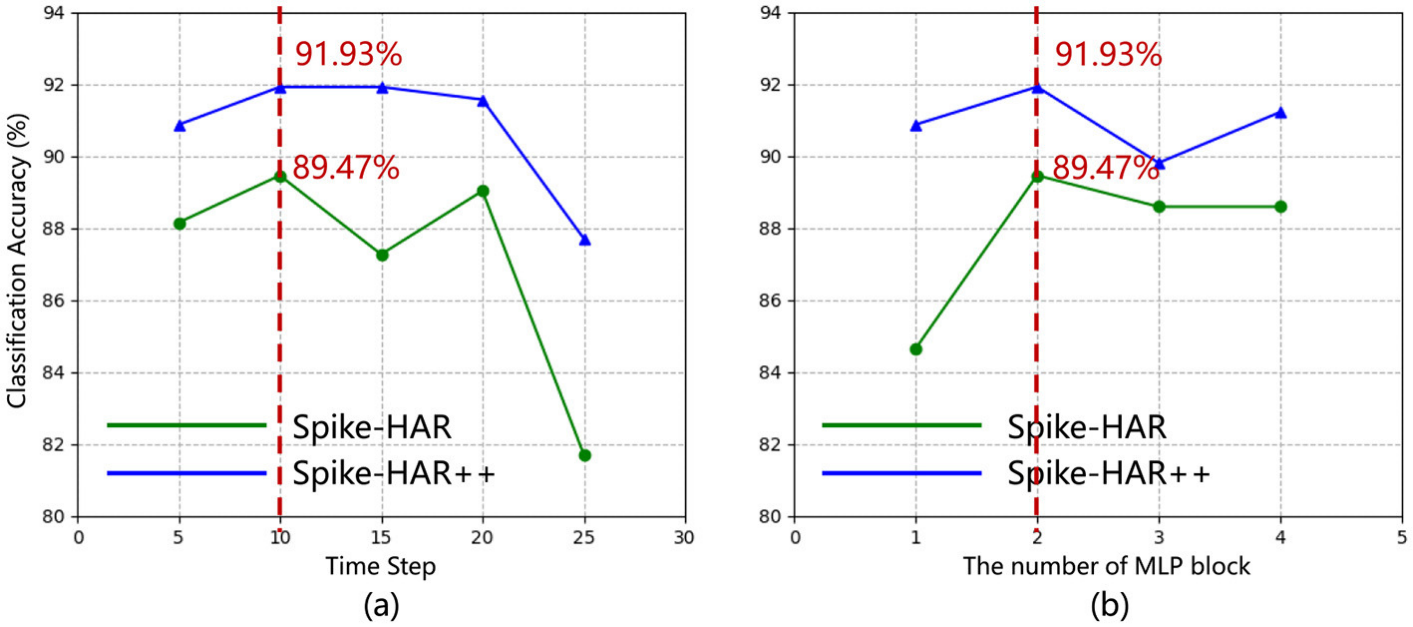
**(a)** Accuracy of Spike-HAR (green line) and Spike-HAR++ (blue line) at varying timesteps *T* (with 2 MLP blocks); **(b)** Accuracy of Spike-HAR (green line) and Spike-HAR++ (blue line) with different numbers of MLP blocks (with *T* = 10).

In this section, we analyze the impact of hyperparameters and the key components of Spike-HAR and Spike-HAR++. Experiments are conducted on the SL-Animals-DVS-4sets dataset. With a fixed total sample length of 500 ms, different time steps are set to investigate the impact of the number of input event frames and transformer blocks on the model results. As can be seen in Figure 4, with the number of time steps and the number of MLP Blocks increasing, the test accuracy of the model does not change significantly, but with the number of time steps increasing to be more than 20 or the number of MLP blocks decreasing to be 1, the test accuracy will have a significant decrease. Specifically, the highest accuracy of 89.47% for Spike-HAR and 91.93% for Spike-HAR++ are achieved by setting the time step to 10 and the number of blocks to 2. On the other hand, the accuracy decreases to 81.72% for Spike-HAR and 87.72% for Spike-HAR++ when the time step is set to 25, and setting the number of blocks to 1 results in an accuracy of 84.65% for Spike-HAR and 90.88% for Spike-HAR++. In addition, The experimental results verify the parallel structure and the attention appearance used in proposed models. As shown in Table 5, using both parallel transformers and attention brunch simultaneously yields the best accuracy in Spike-HAR and Spike-HAR++.

**Table 5:** Accuracy of Spike-HAR and Spike-HAR++ for different architecture on SL-animals-DVS-4sets.

**Models**	**Attention brunch**	**Serial transformer block**	**Parallel transformer block**	**Accuracy**
Spike-HAR		✓		0.8640
	✓	✓		0.8465
			✓	0.8421
	✓		✓	0.8947
Spike-HAR++		✓		0.8640
	✓	✓		0.9088
			✓	0.8421
	✓		✓	0.9193

### 4.5 Energy consumption analysis

We use the SL-Animals-DVS dataset to estimate the energy required for proposed models to classify a DVS sign language video. We first determine the number of operations [SOPs (Zhou et al., 2022) for the SNN module] needed to complete this task:


(14) $$\begin{array}{l} {\mathrm{FLOPs}}_{Conv2D}={\left(k_{n}\right)}^{2}\cdot h_{n}\cdot w_{n}\cdot c_{n-1}\cdot c_{n} \end{array}$$

(15) $$\begin{array}{l} {\mathrm{SOPs}}_{Conv2D}=fr\cdot T_{s}\cdot {\mathrm{FLOPs}}_{Conv2D} \end{array}$$

where *k*_*n*_ is the kernel size, (*t*_*n*_, *h*_*n*_, *w*_*n*_) is the output feature map size, *c*_*n*−1_ and *c*_*n*_ are the input and output channel numbers, respectively. *fr* and *T*_*s*_ denote the spike fire rate and timesteps, respectively. The *fr* is defined as the proportion of non-zero elements within the spike tensor. Practically, we set *T*_*s*_ to 10. Once SOPs for the SNN module are determined, we can further obtain the final energy consumption *E* by multiplying the SOPs with the platform's energy:


(16) $$\begin{array}{l} \;E_{\mathrm{SOPs}}=E_{\mathrm{AC}}\times \mathrm{SOPs} \end{array}$$

We use the same energy efficiency calculation scheme proposed by Hu Y. et al. (2023). The energy consumption is 12.5 pJ for each floating-point operation (FLOP) and is 77 fJ for each synaptic operation (SOP). As shown in Table 6, the Spike-HAR processes DVS frame data with a spatial size of 96 × 96 and a time step of 10 with only 0.03 mJ of power consumption. This represents a 99.27% energy reduction compared to EVT and is substantially lower than that of other baseline models. Furthermore, although Spike-HAR++ has a higher power consumption compared to Spike-HAR (0.06 vs. 0.03 mJ), it is still lower than that of other models and achieves higher performance than Spike-HAR across the SL-Animals-DVS, N-LSA64, DVS128 Gesture, and DailyAction-DVS datasets.

**Table 6:** Computational complexity comparisons of SLR methods.

**Model**	**Method**	**#Params**.	**FLOPs/SOPs**	**Power/mJ**
TORE + ResNet18 (Baldwin et al., 2022)	ANN	11.69 M	3.66 G	45.75
TORE + GoogLeNet (Baldwin et al., 2022)	ANN	8.46 M	2.88 G	36.00
EVT (Sabater et al., 2022)	ANN	0.50 M	0.33 G	4.13
Spike-driven EVT (Yao et al., 2024b)	SNN	66.34 M	6.77 G	0.52
SEW Resnet18 (Fang et al., 2021a)	SNN	2.92 M	1.41 G	0.11
**Spike-HAR (Ours)**	SNN	0.70 M	0.44 G	0.03
**Spike-HAR++ (Ours)**	SNN	1.80 M	0.74 G	0.06

## 5 Conclusion

In this paper, we proprse an energy-efficient and lightweight Spike-HAR family for event-based human action recognition, to adaptively emphasize on local spatial features as well as temporal features. Spike-HAR and Spike-HAR++ surpass existing methods in accuracy on the SL-Animals-DVS, N-LSA64, DVS128 Gesture, and DailyAction-DVS datasets. Furthermore, Spike-HAR and Spike-HAR++ require only 0.03 and 0.06 mJ to recognize a single action event stream, reducing the power consumption of 99.27 and 98.55% compared to the Evt, respectively. It demonstrates the applicability of spiking transformers for human action recognition and their potential application in human-machine interaction and edge HAR devices. In the future, it is promising to develop a more complex large-scale event-based HAR benchmark to further evaluate the performance of the Spike-HAR family in practical applications.

## Data Availability

Publicly available datasets were analyzed in this study. This data can be found here: SL-Animals-DVS (http://www2.imse-cnm.csic.es/neuromorphs/index.php/SL-ANIMALS-DVS-Database); LSA64 (https://facundoq.github.io/datasets/lsa64/); DVS128 Gesture (https://ibm.ent.box.com/s/3hiq58ww1pbbjrinh367ykfdf60xsfm8/folder/50167556794); DailyAction-DVS (https://github.com/qianhuiliu/SNN-action-recognition).
